# Synchrotron radiation analysis of root dentin: the roles of fluoride and calcium ions in hy­droxy­apatite remineralization

**DOI:** 10.1107/S1600577521013655

**Published:** 2022-01-19

**Authors:** Nutthapong Kantrong, Krassawan Khongkhaphet, Nutnicha Sitornsud, Pakaporn Lo-apirukkul, Waraporn Phanprom, Catleya Rojviriya, Penphitcha Amonpattaratkit, Watcharaphong Ariyakriangkai

**Affiliations:** a Department of Restorative Dentistry, Faculty of Dentistry, Khon Kaen University, Khon Kaen, Thailand; b Oral Biology Research Unit, Faculty of Dentistry, Khon Kaen University, Khon Kaen, Thailand; c Research Group of Chronic Inflammatory Oral Diseases and Systemic Disease Associated with Oral Health, Faculty of Dentistry, Khon Kaen University, Khon Kaen, Thailand; d Synchrotron Light Research Institute, Nakhon Ratchasima, Thailand; e Department of Restorative Dentistry and Periodontology, Faculty of Dentistry, Chiang Mai University, Chiang Mai, Thailand

**Keywords:** fluoride, hy­droxy­apatite, root caries, remineralization, synchrotron radiation

## Abstract

Although the use of fluoride for root caries control is reported to be effective, the mechanism of maintaining hy­droxy­apatite is still unclear. This study elucidates the roles of fluoride in the recrystallization of hy­droxy­apatite, and the impact of calcium to maintain the abundance of hy­droxy­apatite on acid-challenged root dentin with a novel approach – using synchrotron radiation. Root dentin samples obtained from 40 extracted human premolars were subjected to pH challenge in combination with fluoride treatment. The effect of fluoride on hy­droxy­apatite regeneration on the root was investigated by using a range of fluoride concentrations (1000–5000 p.p.m.) and the EDTA-chelation technique *in vitro*. Synchrotron radiation X-ray micro-computed tomography and X-ray absorption spectroscopy were utilized to characterize the chemical composition of calcium species on the surface of prepared samples. The percentage of hy­droxy­apatite and the relative abundance of calcium species were subsequently compared between groups. The absence of calcium or fluoride prevented the complete remineralization of hy­droxy­apatite on the surface of early root caries. Different concentrations of fluoride exposure did not affect the relative abundance of hy­droxy­apatite. Sufficient potency of 1000 p.p.m. fluoride solution in promoting hy­droxy­apatite structural recrystallization on the root was demonstrated. Both calcium and fluoride ions are prerequisites in a caries-prone environment. Orchestration of F^−^ and Ca^2+^ is required for structural homeostasis of root dentin during acid attack. Sustainable levels of F^−^ and Ca^2+^ might thus be a strict requirement in the saliva of the population prone to root caries. Fluoride and calcium contribute to structural homeostasis of tooth root, highlighting that routine fluoride use in combination with calcium replenishment is recommended for maintaining dental health. This study also demonstrates that utilization of synchrotron radiation could provide a promising experimental platform for laboratory investigation especially in the dental material research field.

## Abstract 2

Early root carious lesions require an optimal amount of both free fluoride and calcium ions in the oral environment to initiate a hy­droxy­apatite remineralization on root dentin. This study elucidates the role of fluoride and calcium to maintain the abundance of hy­droxy­apatite on acid-challenged root dentin with a novel approach – using synchrotron radiation.

## 1. Introduction

A higher prevalence of root caries has been reported as the number and proportion of people over the age of 65 increases across the globe (Heasman *et al.*, 2017 ▸; UN, 2019 ▸). Considering the causative factors contributing to root caries, such disease is caused by an imbalanced relationship between remineralization and demineralization on the tooth surface (Beltrán-Aguilar *et al.*, 2005 ▸; Young *et al.*, 2015 ▸), a state of which an exchange of calcium, phosphate and fluoride is involved to achieve a saturation point (Neel *et al.*, 2016 ▸). The root portion is also easily approached by cariogenic stimuli due to the thinness of the cementum layer (Dastmalchi *et al.*, 1990 ▸), which puts the underlying dentin at risk of mineral loss. Notably, a previous report has suggested that the formation of fluorapatite during acid attack is not considered as a remineralization since the process involves mineral gain that rather retards further demineralization (Cury & Tenuta, 2009 ▸). Structural deterioration of the tooth root resulting from a chronic polymicrobial infection-induced demineralization process (Young *et al.*, 2015 ▸) thus requires careful structural characterization for a better understanding of the mechanisms of hy­droxy­apatite dissolution where minimally invasive therapy could be implemented for root caries prevention.

Tooth is a hard tissue primarily composed of enamel and dentin with hy­droxy­apatite as the main inorganic component (Zipkin, 1970 ▸). During the formation of hy­droxy­apatite, a diversity of calcium-containing precursors exists for its nucleation, *i.e.* dicalcium phosphate (CaHPO_4_), α- and β-tricalcium phosphate [TCP; Ca_3_(PO_4_)_2_]. The availability of such precursors allows crystallization of hy­droxy­apatite to occur (Francis & Webb, 1970 ▸; Blumenthal & Posner, 1973 ▸). In addition, it has been reported that synthesis of hy­droxy­apatite is achieved by incorporating calcium carbonate (CaCO_3_) and calcium oxide (CaO) in the chemical interactions (Sobczak-Kupiec & Wzorek, 2012 ▸; Kim & Ohtsuki, 2016 ▸; Habibah & Salisbury, 2020 ▸). Notably, hy­droxy­apatite is a form of crystalline calcium phosphate with the formula Ca_10_(PO_4_)_6_(OH)_2_, found in both teeth and bone with slight differences in lattice parameters, crystal size, crystallinity and composition (LeGeros, 1990 ▸).

The use of fluoride has been strongly proven to be effective in preventing dental caries in permanent teeth (SBU, 2002 ▸). Since carious lesions progress more rapidly in dentin than in enamel due to a higher critical pH for dentin and dentin is thus more susceptible to acid dissolution (Kantrong *et al.*, 2021 ▸), prevention of initial root caries has been of utmost importance. In addition, systematic reviews have shown that dentifrice containing 5000 p.p.m. fluoride is more efficient in reducing active root carious lesions when compared with 1100 to 1450 p.p.m. dentifrice, especially for elderly people with exposed root surfaces (Wierichs & Meyer-Lueckel, 2015 ▸). The use of 5000 p.p.m. fluoride toothpaste or gel in preventing root caries has been corroborated by several other investigations (Wierichs & Meyer-Lueckel, 2015 ▸; Slayton *et al.*, 2018 ▸). Most studies have quantified the change in lesion depth, surface microhardness, mineral loss and maximum mineral density due to different levels of fluoride exposure on carious lesions (Shahmoradi *et al.*, 2014 ▸). However, deposition of calcium-based biominerals deposited on the root dentin surface by fluoride is yet unclear. Structural characterization of hy­droxy­apatite regenerated after fluoride application on root dentin hence remains to be investigated.

Previous studies have suggested a prerequisite of free calcium ions (Ca^2+^) in the oral microenvironment for the regeneration of hy­droxy­apatite crystals (Reynolds, 2008 ▸; Chaudhary *et al.*, 2017 ▸). Concentrated fluoride promotes precipitation of calcium and phospho­rus ions over acid-induced early root caries (Kantrong *et al.*, 2021 ▸), possibly suggesting that an ample amount of fluoride is needed for root dentin remineralization. Although the crystalline hy­droxy­apatite in the demineralized and remineralized enamel have been characterized using synchrotron radiation (Asaizumi *et al.*, 2017 ▸; Sui *et al.*, 2018 ▸; Tanaka *et al.*, 2010 ▸), the dynamic change of hy­droxy­apatite and its precursors under acidic circumstances is still unclear, and whether Ca^2+^ and fluoride ions (F^−^) contribute to the hy­droxy­apatite remineralization on the root dentin requires further investigation. Thus, the objectives of this study were to determine the role of fluoride and calcium in hy­droxy­apatite remineralization on early root caries by investigating the gradients of calcium species required for hy­droxy­apatite remineralization. Our analysis implemented using synchrotron radiation as a novel technique to gain a better understanding of how fluoride and calcium ions orchestrate the remineralization of hy­droxy­apatite on root dentin.

## 2. Materials and methods

### 2.1. Preparation of root dentin samples

**Figure 1:**
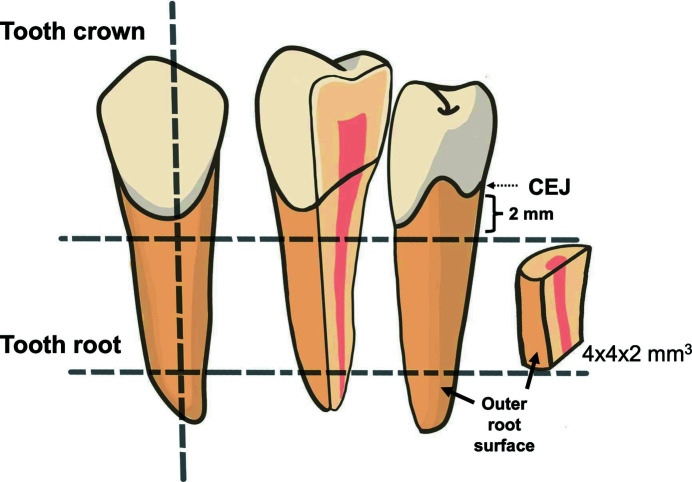
Sectioning of a human premolar to obtain a root dentin specimen for further analysis.

This study was executed under an ethical approval granted by the Ethics Committee for Human Research of Khon Kaen University, based on the declaration of Helsinki (Approval number: HE632048). Briefly, 40 sound human premolar teeth, extracted for orthodontic reasons and stored in 0.1% thymol solution, were cleaned of any residual gingival tissue and cementum using a scalpel blade (No. 15). The teeth were sectioned transversely at a distance of 2 mm underneath the cemento-enamel junction (CEJ) to remove the crown and then longitudinally to produce 4 mm × 4 mm × 2 mm root sections (Fig. 1 ▸) using a precision cutting machine with incorporated water coolant (Mecatome T180 PRESI; Eybens, France). Samples (*N* = 5 per group) were randomly assigned to eight groups as follows:

*Group A* was immersed in deionized water for 11 days serving as the experimental baseline.

*Group B* was immersed in a demineralization solution (2.2 m*M* CaCl_2_, 2.2 m*M* KH_2_PO_4_, 0.05 *M* acetic acid, pH 4.7) for four days to produce early root carious lesions and then immersed in deionized water for the following ten days.

*Groups C, D, E and F* were immersed in a demineralized solution for four days to produce early carious lesions and subjected to a ten-day pH-cycling using the demineralization solution and remineralization solution (1.5 m*M* CaCl_2_, 0.9 m*M* NaH_2_PO_4_, 0.15 *M* KCl, pH 7) along with fluoride treatment solutions containing 0 p.p.m. (*Group C*), 1000 p.p.m. (*Group D*), 1450 p.p.m. (*Group E*) and 5000 p.p.m. fluoride (*Group F*).

*Groups G and H* were subject to the same pH-cycling protocol with an addition of 200 µL of 17% ethyl­enedi­amine tetra­acetic acid (EDTA) to the remineralization solution in order to chelate free Ca^2+^, and treated with 1450 p.p.m. (*Group G*: 1450 p.p.m. F^−^/EDTA) or 0 p.p.m. fluoride solutions (*Group H*: 0 p.p.m. F^−^/EDTA).

### 2.2. pH challenge, treatment with fluoride solution and chelation of Ca^2+^ from remineralization solution

**Figure 2:**
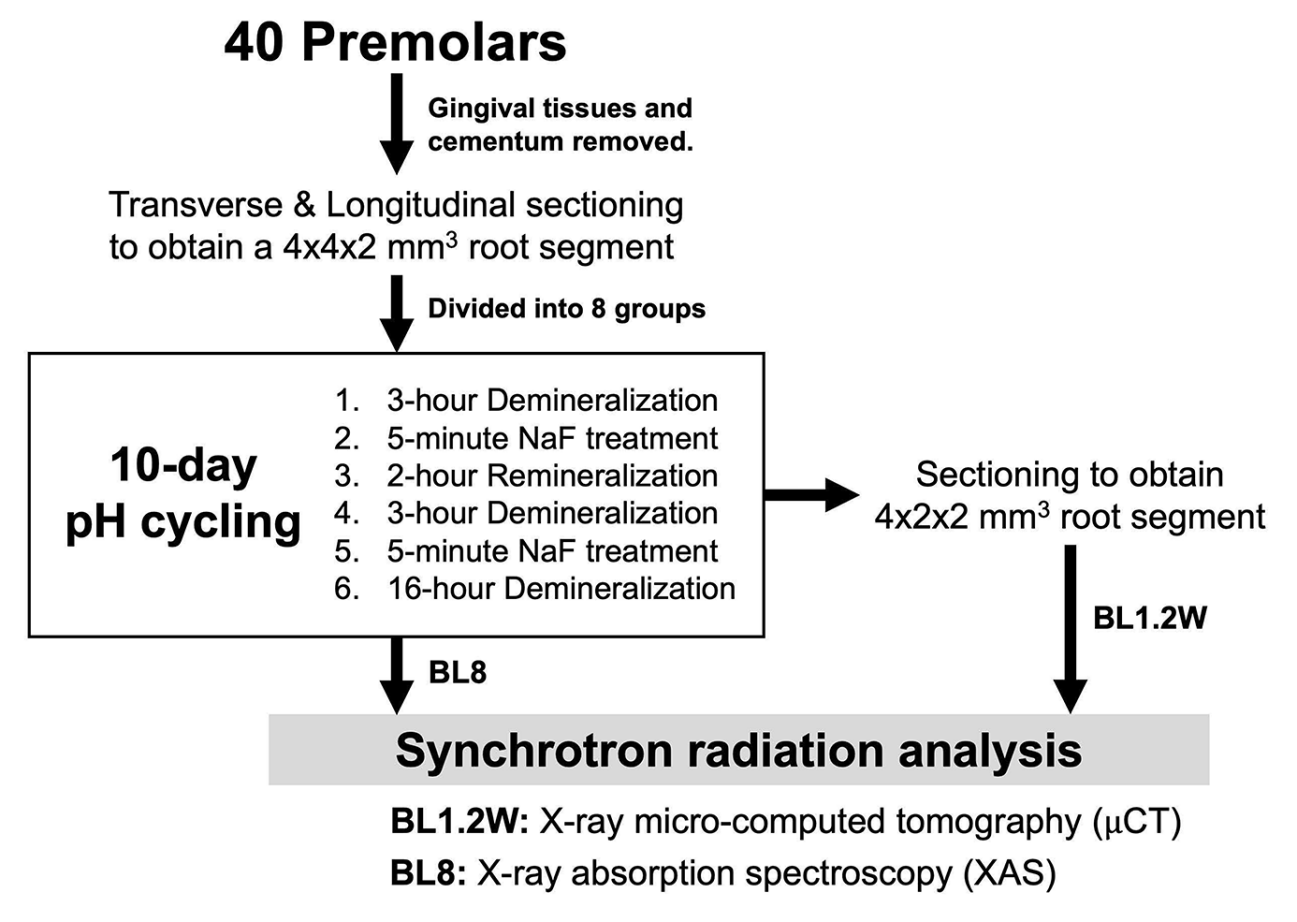
Schematic depiction of laboratory procedures performed in this experimental study.

The pH-cycling was scheduled for ten days in a 37°C environment comprising the following phases (Fig. 2 ▸): 3 h of demineralization twice a day; 5 min sodium fluoride (NaF) solution treatment after each demineralization phase; 2 h of remineralization in between the demineralization phases; and a 16 h overnight period of remineralization.

Treatments were given after each 3 h demineralization period for 5 min. Specimens were immersed in an NaF solution prepared by thoroughly mixing NaF powder (Ricca Chemical, Texas, USA) with the remineralization solution, followed by centrifugation at 100 r.p.m. for 5 min at room temperature. All solutions were prepared daily, and the pH of each solution was monitored to ensure experimental consistency.

**Figure 3:**
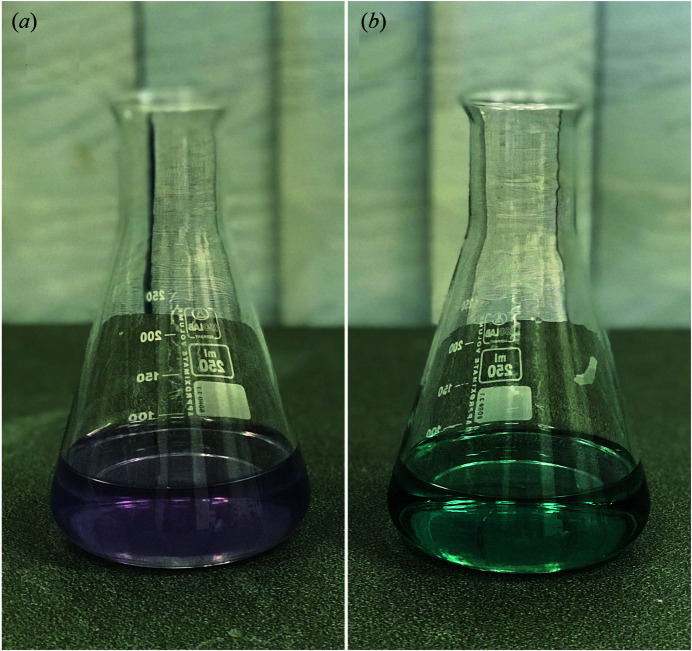
Complexometric titration of free Ca^2+^ in the remineralization solution using 17% EDTA. Note that the colorless remineralization solution turned purple when Eriochrome Black T formed a complex with unbound Ca^2+^ (*a*). However, when 200 µl of 17% EDTA was added, unbound Ca^2+^ formed a complex with EDTA instead and the solution hence immediately turned blue as indicated by Eriochrome Black T. The saturation point of dark blue color indicates the absence of free Ca^2+^ available in the remineralization solution (*b*).

In this study, fluoride solution was chosen for experimental execution as opposed to a dentifrice slurry due to its more precise preparation to obtain the required concentration of fluoride used in a mock experimental treatment. A complexometric titration of remineralization solution with 17% EDTA was used to remove unbound Ca^2+^ to simulate a salivary microenvironment lacking unbound Ca^2+^. The EDTA titration technique was introduced in the early 1960s with the main purpose to determine the calcium level in water and using an Eriochrome Black T as an indicator of the photometric method (Lacy, 1963 ▸; Flaschka & Ganchoff, 1961 ▸). When EDTA formed a complex with Ca^2+^, the solution immediately changed color from purple to blue as indicated by the Eriochrome Black T indicator (Aqua­cheme Co. Ltd., Bangkok, Thailand) (Fig. 3 ▸). A Ca^2+^ chelating end-point was achieved when the remineralization solution became dark blue in color, reflecting the absence of free Ca^2+^. After pH-cycling was completed, the samples were immersed in deionized water; root dentin samples were further dried for 24 h prior to analysis.

Given that the binding ratio of Ca^2+^ to EDTA is 1:1 (Keowmaneechai & McClements, 2002 ▸), 188.5 µL of 17% EDTA was needed for a complete Ca-EDTA chelation in 100 ml remineralization solution. During our preliminary test with complexometric titration, we determined the volume of EDTA used for direct titration and a 200 µl volume of 17% EDTA was sufficient for chelating free Ca^2+^. Eriochrome Black T turned saturated dark blue indicating a complete chemical binding of free Ca^2+^ with EDTA. A volume of 200 µl EDTA in combination with Eriochrome Black T was thus used for freshly preparing the reagents used for the remineralization phase.

### 2.3. Synchrotron radiation X-ray micro-computed tomography

Synchrotron radiation X-ray micro-computed tomography (micro-CT, µCT) was used in this study to non-invasively characterize the three-dimensional structure of dentin specimens (4 mm × 2 mm × 2 mm), and was carried out at beamline BL1.2W at the Synchrotron Light Research Institute (SLRI), Nakhon Ratchasima, Thailand. X-ray radiation was generated from a 2.2 T multipole wiggler at the 1.2 GeV Siam Photon Source. Each specimen was stabilized in a polyimide tube mounted on a rotary stage. A total of 900 X-ray projections were collected for 180° rotation with a step of 0.2°. Images were obtained with a filtered polychromatic X-ray beam at a mean energy of 14 keV and 32 mm distance from the source. All tomographic scans were acquired at a pixel size of 1.44 µm via the detection system equipped with a 200 µm-thick YAG:Ce scintillator, white-beam microscope (Optique Peter, France) and pco.edge 5.5 sCMOS camera (2560 × 2160 pixels, 16 bits). Data processing and tomographic reconstruction were performed using *Octopus Reconstruction* software (Tescan Orsay Holding, Brno, Czech Republic). The 3D visualization of acid-induced root caries was performed using *Drishti* software, Version 2.6 (ANU Vizlab, Canberra, Australia).

### 2.4. Synchrotron radiation analysis of X-ray absorption near-edge structure (XANES)

The calcium *K*-edge was used to characterize the chemical structure of calcium species in carious root dentin using X-ray absortion spectroscoopy (XAS) techniques. The Ca *K*-edge spectra were acquired using fluorescent-yield mode at XAS beamline BL8 of SLRI. The fluorescent-yield spectra of the Ca *K*-edge were obtained from three regions with the following energy ranges and energy steps: 3938.5 to 4018.5 eV (5 eV step), 4018.5 to 4118.5 eV (0.2 eV step) and 4118.5 to 4238.5 eV (5 eV step). The dwell time for each step across all energy regions of samples was 1 s. The fluorescence-yield spectrum was converted to absorption using the following equation (1), routinely used at BL8 SLRI,

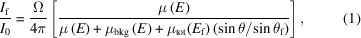

where *I*_0_ is the intensity of the incident X-ray beam, *I*_f_ is the intensity of the monitored fluorescence spectrum, Ω is the solid angle of the detector, *E* is the X-ray energy, *E*_f_ is the energy of the fluorescent X-ray, μ(*E*) is the absorption from the tested element, μ_bkg_(*E*) is the absorption from the background, μ_tot_(*E*) is the total absorption in the sample, θ is the angle of the X-ray on the sample, and θ_f_ is the the angle of emission of the fluorescent X-rays from the sample.

The examined compounds were Ca-based species usually found in hard tissue including hy­droxy­apatite, CaHPO_4_, α-TCP, β-TCP, CaCO_3_ and CaO (Sigma-aldrich, St Louis, MO, USA). The detector used for XAS measurement was a 13-element germanium detector (GeD; Canberra Ultra-LEGe). A digital X-ray processor (XIA DXP-XMAP) on a National Instruments (PXI-1042) crate was used for counting X-ray photons from GeD channels as previously described (Klysubun *et al.*, 2012 ▸). A distinctive absorption edge of each reference compound was used to identify the composition of calcium species in the samples. The absorption spectra were normalized to remove variations between samples. Combinatorial linear combination fitting (LCF) analysis was performed to determine the relative abundance of hy­droxy­apatite and other calcium species by *Athena* software (Ravel & Newville, 2005 ▸) in the energy range 4018.5–4118.5 eV.

### 2.5. Statistical analysis

The mean percentage difference of hy­droxy­apatite was analyzed using a One-way ANOVA with Bonferroni *post hoc* test for multiple comparison among groups. Similar to a previous report (Kantrong *et al.*, 2021 ▸), we utilized the Shannon diversity index which indicates the clustering of specific calcium species on the root to elucidate the role of F^−^ and Ca^2+^ in the environments in hy­droxy­apatite remineralization. Equation (2) was utilized to calculate the Shannon diversity index (Table 1 ▸). Statistical analyses were performed using *GraphPad Prism*, Version 9 (GraphPad Software Inc., La Jolla, CA, USA). A *P*-value of less than 0.05 was considered statistically significant:

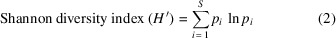

where *S* is the total number of calcium species detected on the surface of the root dentin, and *p_i_* is the proportion of the *i*th calcium species as calculated by dividing the number of the *i*th calcium species by the total number of calcium species detected on the surface of the root dentin.

## 3. Results

### 3.1. Characterization of carious root dentin

**Figure 4:**
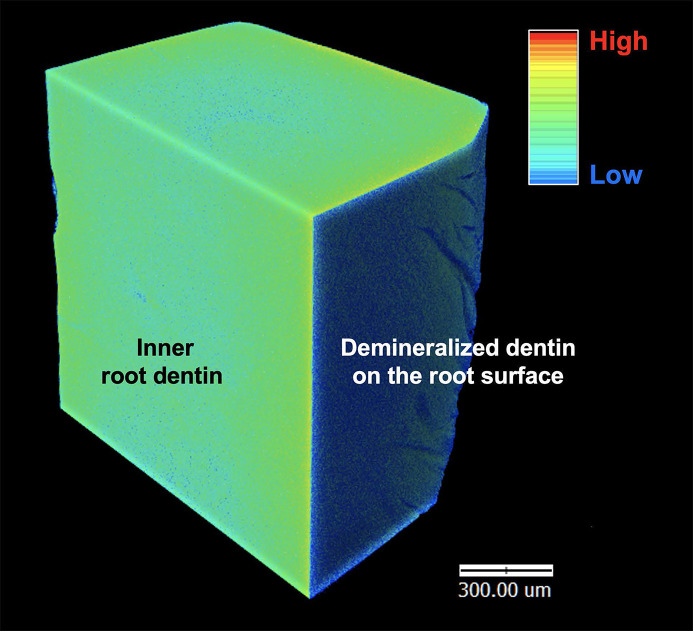
µCT visualization of the demineralized area in the acid-induced group. Induction of surface mineral loss by acidic environment. Colors were assigned according to the density values of each voxel, with higher density values represented by darker colors and low density values by lighter colors.

**Figure 5:**
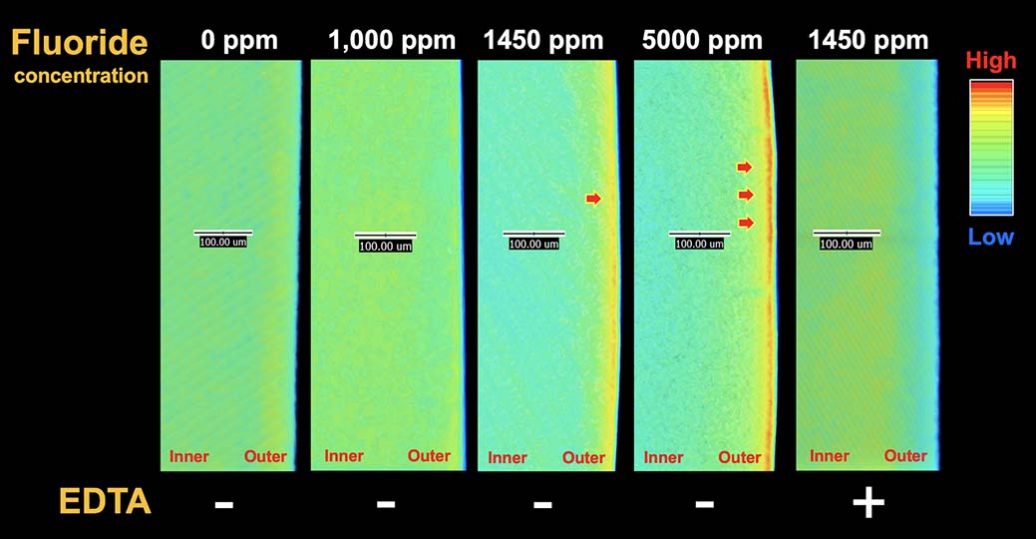
µCT image of superficial mineral deposition on root dentin. Mineral gain on the superficial layer of fluoride-treated samples is clearly seen in a dose-dependent manner as indicated by red arrows.

The formation of early carious root dentin was characterized by using synchrotron radiation µCT. The tomographic volume of the dentin specimens was visualized and analyzed in three dimensions using *Drishti* software. Color gradients were assigned according to the voxel density of the dentin specimen, in which darker colors represent higher density voxels and lighter colors represent lower density voxels. A thin uniform superficial layer, shown in cyan in Fig. 4 ▸, was formed at the surface of root dentin in the acid-induced group. We also noticed a thickening dark band demineralized area on the root dentin when observed under a polarized light microscope (data not shown), indicating the formation of acid-induced early root carious lesions. This result confirmed the process of demineralization at the surface of the specimen by our caries induction protocol. In addition, µCT analysis revealed a dense band of minerals deposited on the root dentin when exposed to fluoride solution, particularly when 1450 p.p.m. and 5000 p.p.m. were used (Fig. 5 ▸).

### 3.2. Hy­droxy­apatite is the main component of root dentin

**Figure 6:**
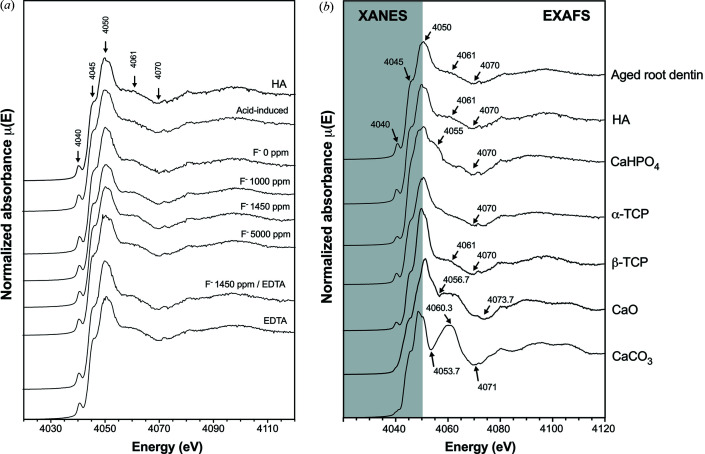
Spectral features of XAS consisting of X-ray absorption near-edge structure (XANES) specifically used for structural analysis and extended X-ray absorption fine structure (EXAFS). (*a*) The Ca-absorption edge obtained for each treatment group. (*b*) The structures in the absorption edge of the aged root dentin group strongly resemble the features found in the absorption edge of hy­droxy­apatite, indicating that hy­droxy­apatite is the main calcium species found in aged root dentin.

**Figure 7:**
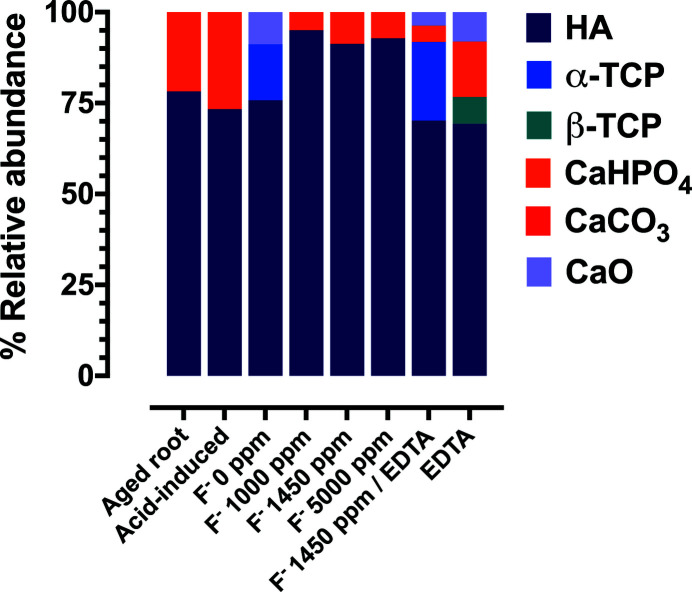
The diversity shift of calcium derivatives in the presence of F^−^ and Ca^2+^ in the environment. Relative abundance (%) of the following calcium species: hy­droxy­apatite, α-TCP, β-TCP, CaHPO_4_, CaCO_3_, CaO found in each treatment group.

Characterization of the mineral composition of the samples was performed by the acquisition of XANES [Fig. 6 ▸(*a*)] using XAS techniques. The structures in the absorption edge of the samples were compared with known spectra of calcium species in human hard tissue. We found that the white line and shoulder of the aged root dentin samples strongly resembled the features found in the absorption edge of hy­droxy­apatite [Fig. 6 ▸(*b*)]. LCF was subsequently performed to determine the most likely chemical composition of each root dentin specimen. We found that aged root dentin is composed of 78.2% hy­droxy­apatite and 21.8% CaHPO_4_ (Fig. 7 ▸, Table 1 ▸). The *R*-factor value, which indicates the fit of the LCF analysis, was in the range <0.002–0.04 (Table 1 ▸), suggesting a high fitting quality. This reaffirmed that hy­droxy­apatite is the main native calcium species detected on root dentin.

### 3.3. Fluoride promotes hy­droxy­apatite recrystallization on root dentin

In this study, we sought to determine whether fluoride solutions affected the remineralization pattern of calcium species on carious root lesions. XAS results indicate that a statistically significant difference (*P*-value < 0.0001) of 73.30% relative abundance of hy­droxy­apatite in the 0 p.p.m. fluoride group compared with 95.0% in the 1000 p.p.m. fluoride group was present (Fig. 7 ▸, Table 2 ▸). It is worth noting that the diversity of calcium species found in the treated root dentin were increased in the absence of F^−^, as indicated by the Shannon diversity index, when comparing the fluoride-untreated group (0 p.p.m. fluoride, *H*′ = 0.7120) with the fluoride-treated group (1000 p.p.m., *H*′ = 0.1985; 1450 p.p.m., *H*′ = 0.2955; 5000 p.p.m., *H*′ = 0.2588). This suggested that the presence of F^−^ favors the transformation of hy­droxy­apatite precursors, detected in the environment devoid of fluoride, to other calcium species. Under the condition containing F^−^, clustering of hy­droxy­apatite from the transitioning of other calcium species was detected as indicated by a decreased Shannon diversity index calculated from those groups treated with F^−^.

### 3.4. Hy­droxy­apatite remineralization requires free Ca^2+^ in the remineralization condition

As analyzed by XAS, the relative abundance of hy­droxy­apatite in the 1450 p.p.m. fluoride group and 1450 p.p.m. fluoride group with EDTA were compared to determine the role of Ca^2+^ in hy­droxy­apatite remineralization. The 1450 p.p.m. group with EDTA demonstrated a statistically significant difference (*P*-value < 0.0001) of 70.2% relative abundance of hy­droxy­apatite compared with 91.3% in the 1450 p.p.m. group (Table 2 ▸). In the environment containing 1450 p.p.m. fluoride, the diversity index (*H*′ = 0.2955) was lower than that of the calcium ion-deprived environment in the 1450 p.p.m. fluoride with EDTA (*H*′ = 0.8409). This discrepancy indicates that complete remineralization of hy­droxy­apatite requires free, unbound Ca^2+^ in the oral microenvironment in order to achieve the optimal aqueous condition needed for a remineralization to prevail.

### 3.5. Contribution of Ca^2+^ and F^−^ on the hy­droxy­apatite remineralization on early carious root dentin

To elucidate the role of Ca^2+^ and F^−^ in the remineralization process of carious root dentin, the relative abundance of hy­droxy­apatite in the acid-induced group and EDTA group were compared. It is worth noting that, in the absence of fluoride, CaO was detected and the diversity of calcium species was increased (*H*′ = 0.7120) when compared with the acid-induced group (*H*′ = 0.5803). In addition, there was no statistical significance (*P*-value > 0.05, Table 2 ▸) when comparing the percentage of hy­droxy­apatite in both groups, indicating that the relative abundance of the dominant calcium species remained unchanged in the absence of F^−^. Similar to the 1450 p.p.m. fluoride group with EDTA, there was no intense superficial band on the root formed in the 0 p.p.m. fluoride group with EDTA (Fig. 5 ▸). The percentage of hy­droxy­apatite regenerated in a 1450 p.p.m. fluoride group with EDTA (70.2%) and a 0 p.p.m. fluoride group with EDTA (69.3%) was not statistically different (*P*-value > 0.05, Table 2 ▸). However, a slight increase of the Shannon diversity index was found in a group of 0 p.p.m. fluoride with EDTA (*H*′ = 0.9367) when compared with a group of 1450 p.p.m. fluoride with EDTA (*H*′ = 0.8409). β-TCP was also present in a group of 0 p.p.m. fluoride with EDTA which lacked both F^−^ and Ca^2+^, indicating that nucleation of calcium species on the root was diversified in the environment devoid of free F^−^ and Ca^2+^.

The groups with 1000 p.p.m., 1450 p.p.m. and 5000 p.p.m. fluoride solutions were used to determine the effect of different concentrations of fluoride solutions on the relative abundance of remineralized hy­droxy­apatite. The relative abundance of hy­droxy­apatite across all three groups was statistically insignificant (*P*-value > 0.05) when compared with 0 p.p.m. fluoride (Table 2 ▸). Different concentrations of fluoride solutions had no effect on the relative abundance of remineralized hy­droxy­apatite, suggesting a robust effect of fluoride application on hy­droxy­apatite recrystallization, even at the 1000 p.p.m. fluoride concentration.

## 4. Discussion

Despite the significance of fluoride use as a non-invasive method in managing root carious lesions being extensively addressed (Cai *et al.*, 2018 ▸), it is still controversial as to which concentration of fluoride is sufficient to achieve an optimal level of mineral regain. Thus, this is the first study investigating the fluoride-induced remineralization of hy­droxy­apatite by means of chemical structural analysis. Utilization of synchrotron radiation in dental biomaterial research has allowed us to closely elucidate the effect of fluoride in promoting hy­droxy­apatite crystal regeneration by means of a non-invasive strategy to gain a better understanding of how the mechanism of protection against root hy­droxy­apatite dissolution occurs in the presence of fluoride. In support of the use of synchrotron radiation, recent studies have demonstrated its potential for biomedical application including synchrotron radiation XAS for a precise cellular elemental localization as well as the identification of calcium compounds using XANES analysis in human cartilage (Nguyen *et al.*, 2011 ▸; Ortega *et al.*, 2009 ▸). Thus, an analysis of dental hard tissue and dental biomaterials using synchrotron radiation could be a laboratory strategy of choice that holds great promise for specific target structural detection with high sensitivity.

Acid produced by bacteria in dental plaque causes the dissolution of calcium and phosphate ions from apatite crystals (LeGeros, 1990 ▸). Our findings demonstrated the accumulation of CaHPO_4_ in addition to hy­droxy­apatite when root dentin was acid attacked. It is possible that a slight amount of hy­droxy­apatite was dissolved thereby increasing the deposition of CaHPO_4_ during an acid attack. Interestingly, TCP had not yet been recovered from dissolved hy­droxy­apatite possibly owing to the relatively higher solubility product constant (*K*_sp_) of TCP species when compared with hy­droxy­apatite rendering hy­droxy­apatite the most stable form of calcium species examined (Chow, 2009 ▸). However, surface mineral loss is expected to have occurred in terms of quantity during the acid challenge (Wierichs *et al.*, 2020 ▸). This is in part explained by a decreased deposition of hy­droxy­apatite in acid-induced specimens. Interestingly, the presence of CaHPO_4_ has suggested that it may possibly serve as a calcium reservoir required for hy­droxy­apatite nucleation on the root dentin.

Even though remineralization can occur even in the absence of fluoride in oral microenvironments (ten Cate, 1999 ▸), a treatment without F^−^ resulted in a lower abundance of hy­droxy­apatite when compared with fluoride treatment. This suggested that F^−^ specifically promotes hy­droxy­apatite remineralization of root dentin after a cariogenic attack. It is worth addressing that increasing concentrations of fluoride did not affect the relative abundance of hy­droxy­apatite formed. The relatively unchanged abundance of hy­droxy­apatite across environments with varying concentrations of fluoride was possibly due to the reached equilibrium between the remineralization of hy­droxy­apatite and the transformation of hy­droxy­apatite into fluoride species (Cury & Tenuta, 2009 ▸; Li *et al.*, 2014 ▸). Our µCT analysis also revealed a dense band of deposited mineral on the root when 1450 p.p.m. and 5000 p.p.m. fluoride were applied, possibly indicating the formation of fluoride-containing calcium species. However, higher fluoride content in the environments markedly decrease the size of crystallites (Asaizumi *et al.*, 2017 ▸). Further quantification of fluoride-containing calcium species and characterization of the apatite crystal sizes are needed to corroborate the effect of fluoride use on fluorapatite deposition on root dentin in order to gain insight into how root dentin achieves resistance against acid challenge, due to the presence of a protective layer of acid-resistant fluoride species (McCann, 1968 ▸; Crommelin *et al.*, 1983 ▸).

The loss of Ca^2+^ and PO_4_^3−^ is recovered when the biofilm is supersaturated with hy­droxy­apatite and fluorapatite at pH above 5.5, suggesting that hy­droxy­apatite regeneration occurs concomitant with fluorapatite gain (Cury & Tenuta, 2009 ▸). Our findings suggested that the absence of F^−^ in a Ca^2+^-rich environment results in a redeposition of α-TCP, a hy­droxy­apatite precursor, indicating that F^−^ is required for promoting hy­droxy­apatite crystal maturation. When Ca^2+^ was removed by the EDTA chelation technique, a remineralization of hydroxy­apatite failed to occur as indicated by the accumulation of α-TCP, CaHPO_4_ and CaO, which are known precursors of hy­droxy­apatite (Habibah & Salisbury, 2020 ▸). Interestingly, CaCO_3_ was not detected which may suggest the dependence of hy­droxy­apatite recrystallization on the availability of Ca^2+^ and PO_4_^3−^ ions, not on carbonate ions. Perhaps carbonate apatite is formed but the high solubility of carbonate in an acidic microenvironment may lead to its absence on tooth substrates (Neel *et al.*, 2016 ▸).

In an environment devoid of both F^−^ and Ca^2+^, β-TCP was formed rather than α-TCP. The lack of F^−^ and Ca^2+^ might allow the shift of hy­droxy­apatite on root dentin to β-TCP, which is a more stable form when compared with α-TCP (Cohn *et al.*, 2017 ▸), to redeposit on root dentin. Undersaturation of F^−^ in the milieu might thus fail to induce a complete hy­droxy­apatite recrystallization, and the effect was even more pronounced in the environment lacking both F^−^ and Ca^2+^. Despite the presence of F^−^, the lack of Ca^2+^ in the environment might result in the incomplete transformation of hy­droxy­apatite precursors, *i.e.* α-TCP, CaHPO_4_ and CaO into mature hy­droxy­apatite crystals. Although root dentin consists of calcium-containing precursors of hy­droxy­apatite, our results indicate that remineralization on root dentin is enhanced by unbound Ca^2+^, and the transformation of existing calcium precursors on the root dentin to mature hy­droxy­apatite is driven by F^−^ in the environment. Taken together, our data indicated that Ca^2+^ and F^−^ in the environment contribute to hy­droxy­apatite nucleation. The clinical implications of this applies directly to xerostomic patients or patients receiving radiotherapy in the head and neck region, who may have limited salivary calcium and phosphate levels. This underscores the use of calcium-containing products such as casein phospho­peptide–amorphous calcium phosphate (CPP–ACP) in replenishing depleted oral calcium reservoirs, to possibly facilitate fluoride-mediated hy­droxy­apatite recrystallization (Rose, 2000 ▸). Use of fluoride-containing agents in conjunction with a topical CPP–ACP might allow a greater amount of hy­droxy­apatite recrystallization to occur in patients with high root caries risk, and also decreased toxicity when high dose fluoride is used.

In addition to hy­droxy­apatite, precursors of hy­droxy­apatite remained present as indicated in the aged root dentin group. A combination of β-TCP and fluoride are known to enhance the remineralization process (Arifa *et al.*, 2019 ▸). This reaction can be used to explain the absence of TCP in the fluoride-treated samples where both Ca^2+^ and F^−^ were present. This reaffirms that the presence of F^−^ drives the transition of tricalcium phosphate into fluoride species (Li *et al.*, 2014 ▸). The presence of CaHPO_4_, instead of TCP in the acid-induced and EDTA groups, can be attributed to the higher *K*_sp_ of CaHPO_4_ (Chow, 2009 ▸). Herein, our laboratory analysis demonstrated a beneficial use of synchrotron radiation as an alternative method for dental biomaterial research analysis which could provide an accurate structural characterization under a high-throughput analysis using synchrotron-based XAS. Further investigation on formed fluoride-containing calcium species in the microenvironment containing salivary components *in vivo* is needed to provide insight on root mineral gain and aid in the rational use of fluoride to yield efficient root dentin protection under low concentration used. However, carbonated apatite structure has been identified and the nonstoichiometric forms of apatite are believed to be major components constituting human bone and teeth (Frank-Kamenetskaya, 2008 ▸; Leventouri *et al.*, 2009 ▸). Further elucidation of the apatite-like structural deposition is needed for a better insight on natural remineralization of the nonstoichiometric apatite on root dentin as promoted by F^−^ and Ca^2+^. To the best of our knowledge within the technical limitation of this study employing an artificial system, our results first however explained the dynamic basis of chemical hy­droxy­apatite dissolution from root dentin, in relation to its precursors. With hy­droxy­apatite being the dominant calcium species, understanding the dynamic change of hy­droxy­apatite remineralization in root dentin has implications on the prevention of root caries by targeting a specific mechanism required for maintaining structural homeostasis.

## 5. Conclusion

In summary, our study is the first laboratory quantitative report on the chemical gradient of calcium compounds associated with the remineralization of root dentin hy­droxy­apatite as analyzed by synchrotron radiation analysis – a novel qualitative approach. This study sheds light on the chemical aspect of hy­droxy­apatite remineralization on the root dentin by demonstrating that F^−^ and Ca^2+^ are essential requirements and that the availability of calcium and phosphate ions is also vital for this process. Fluoride promotes a specific clustering of hy­droxy­apatite deposited on the root, regardless of the fluoride concentration used. The use of calcium phosphate products in tandem with fluoride for managing early root carious lesions is thus recommended.

## Figures and Tables

**Table 1:** The percentage and standard deviation of investigated calcium species in each treatment group and calculated Shannon diversity index

Intervention	Hy­droxy­apatite		α-TCP	β-TCP	CaHPO_4_	CaCO_3_	CaO	*R*-factor	Shannon diversity index (*H*′)
A: Aged root dentin	78.2 ± 3.7		0	0	21.8 ± 2.4	0	0	0.041559	0.5244
B: Acid-induced	73.3 ± 3.8		0	0	26.7 ± 6.3	0	0	0.007244	0.5803
C: F 0 p.p.m.	75.8 ± 4.9		15.4 ± 4.0	0	0	0	8.8 ± 1.8	0.0038061	0.7120
D: F 1000 p.p.m.	95.0 ± 2.6		0	0	5.0 ± 5.3	0	0	0.0045462	0.1985
E: F 1450 p.p.m.	91.3 ± 1.8		0	0	8.7 ± 4.0	0	0	0.0021873	0.2955
F: F 5000 p.p.m.	92.8 ± 2.0		0	0	7.2 ± 2.0	0	0	0.0033991	0.2588
G: F 1450 p.p.m./EDTA	70.2 ± 2.3		21.6 ± 3.8	0	4.5 ± 5.8	0	3.7 ± 1.5	0.0020471	0.8409
H: F 0 p.p.m./EDTA	69.3 ± 3.3		0	7.4 ± 2.1	15.2 ± 3.0	0	8.1 ± 5.5	0.0015962	0.9367

**Table 2:** Comparison of the mean of hy­droxy­apatite percentage in terms of relative abundance for each treatment group Asterisks (*) indicate a significant difference. Ns = non-significant.

Intervention	*P*-value	Summary	Group
Acid-induced / F 0 p.p.m.	0.0580	Ns	*B–C
Acid-induced / F 1000 p.p.m.	<0.0001	Significant	*B–D
Acid-induced / F 1450 p.p.m.	<0.0001	Significant	*B–E
Acid-induced / F 5000 p.p.m.	<0.0001	Significant	*B–F
Acid-induced / F 1450 p.p.m. with EDTA	>0.9999	Ns	B–G
Acid-induced / F 0 p.p.m. with EDTA	>0.9999	Ns	B–H
F 0 p.p.m. / F 1000 p.p.m.	<0.0001	Significant	*C–D
F 0 p.p.m. / F 1450 p.p.m.	<0.0001	Significant	*C–E
F 0 p.p.m. / F 5000 p.p.m.	<0.0001	Significant	*C–F
F 0 p.p.m. / F 1450 p.p.m. with EDTA	0.1798	Ns	C–G
F 0 p.p.m. / F 0 p.p.m. with EDTA	0.0580	Ns	C–H
F 1000 p.p.m. / F 1450 p.p.m.	>0.9999	Ns	D–E
F 1000 p.p.m. / F 5000 p.p.m.	>0.9999	Ns	D–F
F 1000 p.p.m. / F 1450 p.p.m. with EDTA	<0.0001	Significant	*D–G
F 1000 p.p.m. / F 0 p.p.m. with EDTA	<0.0001	Significant	*D–H
F 1450 p.p.m. / F 5000 p.p.m.	>0.9999	Ns	E–F
F 1450 p.p.m. / F 1450 p.p.m. with EDTA	<0.0001	Significant	*E–G
F 1450 p.p.m. / F 0 p.p.m. with EDTA	<0.0001	Significant	*E–H
F 5000 p.p.m. / F 1450 p.p.m. with EDTA	<0.0001	Significant	*F–G
F 5000 p.p.m. / F 0 p.p.m. with EDTA	<0.0001	Significant	*F–H
F 1450 p.p.m. with EDTA / F 0 p.p.m. with EDTA	>0.9999	Ns	G–H
